# Connection of Cancer Exosomal LncRNAs, Sponging miRNAs, and Exosomal Processing and Their Potential Modulation by Natural Products

**DOI:** 10.3390/cancers15082215

**Published:** 2023-04-09

**Authors:** Ya-Ting Chuang, Jun-Ping Shiau, Jen-Yang Tang, Ammad Ahmad Farooqi, Fang-Rong Chang, Yi-Hong Tsai, Ching-Yu Yen, Hsueh-Wei Chang

**Affiliations:** 1 Graduate Institute of Medicine, College of Medicine, Kaohsiung Medical University, Kaohsiung 80708, Taiwan; u111500006@gap.kmu.edu.tw; 2 Division of Breast Oncology and Surgery, Department of Surgery, Kaohsiung Medical University Hospital, Kaohsiung Medical University, Kaohsiung 80708, Taiwan; 1060526@kmuh.org.tw; 3 School of Post-Baccalaureate Medicine, Kaohsiung Medical University, Kaohsiung 80708, Taiwan; reyata@kmu.edu.tw; 4 Department of Radiation Oncology, Kaohsiung Medical University Hospital, Kaohsiung Medical University, Kaohsiung 80708, Taiwan; 5 Institute of Biomedical and Genetic Engineering (IBGE), Islamabad 54000, Pakistan; farooqiammadahmad@gmail.com; 6 Graduate Institute of Natural Products, Kaohsiung Medical University, Kaohsiung 80708, Taiwan; aaronfrc@kmu.edu.tw (F.-R.C.); r960134@kmu.edu.tw (Y.-H.T.); 7 School of Dentistry, Taipei Medical University, Taipei 11031, Taiwan; 8 Department of Oral and Maxillofacial Surgery, Chi-Mei Medical Center, Tainan 71004, Taiwan; 9 Department of Biomedical Science and Environmental Biology, College of Life Science, Kaohsiung Medical University, Kaohsiung 80708, Taiwan; 10 Center for Cancer Research, Kaohsiung Medical University, Kaohsiung 80708, Taiwan

**Keywords:** exosome, anticancer, lncRNAs, sponging miRNAs, exosomal processing, natural product

## Abstract

**Simple Summary:**

Cancer cells generally release special vesicles (exosomes) showing tumor-promoting effects. Some natural products blocking exosome processing (assembly and secretion) can inhibit cancer progression. Long noncoding RNAs (lncRNAs), enclosed in exosomes, can bind and modulate several microRNAs (miRNAs), and, in turn, miRNAs can regulate their targets, such as exosome processing genes. However, there is a gap in the correlation between exosomal lncRNAs and exosomal processing of natural product treatments. After collecting and organizing literature reports, we introduce bioinformatics for retrieving miRNA targets of lncRNAs and exosomal processing gene targets of miRNAs to fill this gap. Consequently, the function of exosomal lncRNAs of cancer cells in regulating miRNA targets that potentially modulate exosomal processing genes is summarized, particularly for the anticancer effects of natural products.

**Abstract:**

Cancerous exosomes contain diverse biomolecules that regulate cancer progression. Modulating exosome biogenesis with clinical drugs has become an effective strategy for cancer therapy. Suppressing exosomal processing (assembly and secretion) may block exosomal function to reduce the proliferation of cancer cells. However, the information on natural products that modulate cancer exosomes lacks systemic organization, particularly for exosomal long noncoding RNAs (lncRNAs). There is a gap in the connection between exosomal lncRNAs and exosomal processing. This review introduces the database (LncTarD) to explore the potential of exosomal lncRNAs and their sponging miRNAs. The names of sponging miRNAs were transferred to the database (miRDB) for the target prediction of exosomal processing genes. Moreover, the impacts of lncRNAs, sponging miRNAs, and exosomal processing on the tumor microenvironment (TME) and natural-product-modulating anticancer effects were then retrieved and organized. This review sheds light on the functions of exosomal lncRNAs, sponging miRNAs, and exosomal processing in anticancer processes. It also provides future directions for the application of natural products when regulating cancerous exosomal lncRNAs.

## 1. Introduction

Exosomes are nano-sized extracellular vesicles. Many protein and lipid biomolecules and nucleic acid molecules, such as DNAs, mRNAs, and noncoding RNAs, are generally included in exosomes [1,2,3]. Several exosomal noncoding RNAs (ncRNAs) have been reported, including circular RNAs, long noncoding RNAs (lncRNAs), and microRNAs (miRNAs) [1,2,3]. The secretion of exosomes is higher in cancer than in normal cells [3,4,5,6,7,8,9,10,11,12], suggesting that cancer exosomes play a crucial role in regulating cancer cell progression such as proliferation [1,2,13], drug resistance [1,14], migration/invasion [15,16], metastasis [17], and tumor microenvironments (TME) [18,19,20].

Exosome generation includes exosomal assembly and secretion. Exosomal assembly is initiated from endocytosis. It continuously forms early and late endosomes and matures to form the multivesicular body (MVB). During exosomal assembly, the components of exosomes are uploaded. Then, exosomal secretion is activated by fusing the MVB with the plasma membrane for exocytosis [21,22,23,24,25,26,27,28,29]. Exosomal secretion and assembly signaling was reported from the Mouse Genome Database in Gene Oncology (GO:1990182) [30].

As mentioned, in the Gene Oncology of the Mouse Genome Database [30] (https://www.informatics.jax.org/vocab/gene_ontology/GO:0071971) (access on 1 March 2023), a set of proteins are reported to regulate exosomal assembly, including the CD34 antigen (CD34), programmed cell death 6 interacting protein (PDCD6IP), syndecan 1 (SCD1), SDC4, syndecan-binding protein (SDCBP), SH3 domain and ITAM motif (STAM), and tumor susceptibility gene 101 (TSG101) [30]. In comparison, several proteins modulate exosomal secretion, including the COP9 signalosome subunit 5 (COPS5), RAB11A, a member of the RAS oncogene family (RAB11A), RAB7A, RAB7B, RAB27A, STEAP family member 3 (STEAP3), ATPase class II, type 9A (ATP9A), ATP13A2, parkin RBR E3 ubiquitin protein ligase (PRKN), vacuolar protein sorting 4A (VPS4A), VPS4B, charged multivesicular body protein 2A (CHMP2A), hepatocyte growth factor-regulated tyrosine kinase substrate (HGS), MYO5B, PDCD6IP, SDC1, SDC4, SDCBP, sphingomyelin phosphodiesterase 3, neutral (SMPD3), SNF8 subunit of the endosomal sorting complexes required for transport (ESCRT)-II complex (SNF8), STAM, and TSG101. Some exosomal assembly and secretion genes overlap, such as PDCD6IP, SDC1, SDC4, SDCBP, STAM, and TSG101 [30].

Although many exosomal ncRNAs are essential for participating in carcinogenesis [31], the present review focuses on lncRNAs. Noncoding transcripts over 200 nucleotides are called lncRNAs. Mounting literature reports show that exosomal lncRNAs control cancer progression, such as lung [32], gynecological [11], gastric [33], and other cancers. Different lncRNAs exhibit diverse functions. The regulatory mechanisms of lncRNAs were collected in the database LncTarD [34] and include transcriptional regulation, ceRNAing or miRNA sponging, chromatin looping, epigenetic regulation (histone modification and DNA methylation), interacting with mRNA (mRNA splicing and RNA editing), and interacting with protein (protein stability and phosphorylation). LncRNAs have multiple regulatory mechanisms. Each lncRNA, in turn, modulates several miRNAs or proteins transcriptionally and epigenetically, demonstrating a complex network and making it difficult to provide a straightforward direction and clear organization.

**Figure 1:**
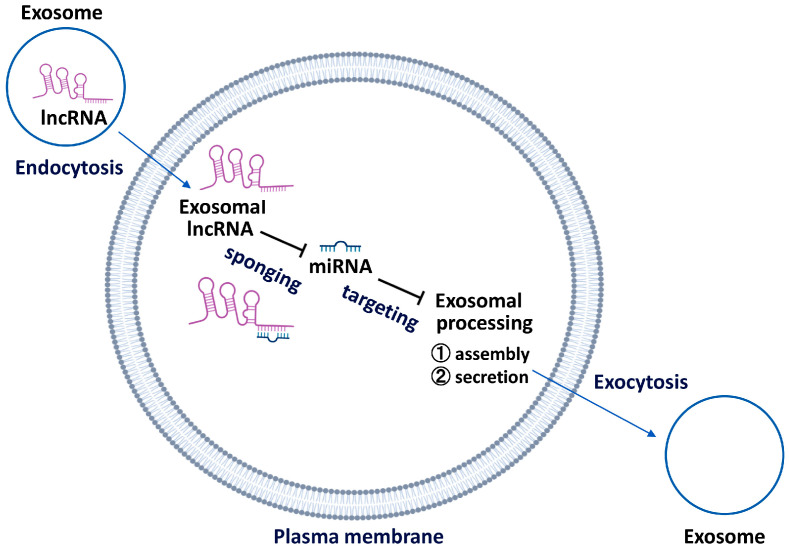
Overview of the objectives of this review. The lncRNA–miRNAs–exosomal processing targets axis is shown. It starts from exosomal lncRNAs sponging miRNAs that target exosomal processing (including (1) assembly and (2) secretion) genes. For clarification, the detailed components of exosomes are not shown, and only the lncRNAs and miRNAs are indicated.

For simplification, the present review focuses on the exosomal lncRNAs that may exhibit miRNA-binding sites to sponge miRNAs, causing specific miRNA degradation and preventing them from binding their target mRNAs [35,36] (Figure 1). MiRNAs are another group of ncRNAs with short lengths (21–25 nucleotides). The function of miRNAs is the direct regulation of mRNA degradation and indirect modulation of protein expression by blocking translation [37].

Notably, a single miRNA potentially targets several hundred genes. A literature survey, starting from cancer exosomal lncRNAs and continuing to sponging miRNAs and connecting to their target genes, suggests their effects to be similar to a signaling cascade. To converge the connection of exosomal lncRNA-sponging miRNA targets, the targets of the genes involved in exosomal processing were chosen as described above. Moreover, the relationship between miRNAs and exosomal processing genes is poorly known at present. Therefore, in the present review, we focus on the exosomal lncRNA–sponging miRNA–exosomal processing target axis (Figure 1).

**Figure 2:**
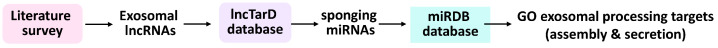
Steps in filling the knowledge gap between exosomal lncRNA and sponging miRNAs and between miRNAs and exosomal processing targets, as provided by lncTarD [34] and miRDB [38] databases.

Exosomal lncRNAs are overexpressed in cancer and responsible for cancer progression. The strategy of downregulating exosomal lncRNAs has potential in anticancer therapy. Some natural products exhibit anticancer effects that may be attributed to suppressing the exosomal lncRNAs of cancer. However, the potential sponging miRNAs and exosomal processing targets of exosomal lncRNAs are rarely summarized. The gaps between exosomal lncRNA and sponging miRNAs and between miRNAs and exosomal processing genes are filled by bioinformatic tools, such as lncTarD [34] and miRDB [38], respectively (Figure 2).

For this review, we collected several cancer exosomal lncRNAs and performed bioinformatic retrieval for their sponging miRNAs and functions and the targeting of exosomal processing genes (Section 2). Next, tumor microenvironment (TME)-associated lncRNAs, sponging miRNAs, and exosomal processing genes were assessed (Section 3). Finally, the lncRNA-modulating effects of natural products, their sponging action on miRNAs, and the exosomal processing of cancer cells were explored (Section 4). This review examines the relationship between cancer exosomal lncRNAs, miRNAs, and the exosomal processing targets of cancer cells as well as the potential impact of natural products on this axis. Compared to previous literature reports, as mentioned later, this review provides a novel integration for each function of natural-product-modulating exosomal lncRNAs, miRNAs, and the exosomal processing targets by filling the connecting gap with bioinformatic databases.

## 2. Cancer Exosomal lncRNAs, Sponging miRNAs, and Exosome Processing (Secretion and Assembly)

Many exosomal lncRNAs have been reported to be overexpressed in cancer cells [32,39,40,41,42,43,44,45,46,47,48,49]. However, their potential sponging miRNAs are rarely reported. In this review, the sponging miRNAs of exosomal lncRNAs were bioinformatically identified using the lncTarD database [34]. Moreover, the sponging-miRNA-targeting putative exosomal processing genes were bioinformatically retrieved using the miRDB database [38], as shown in the following.

### 2.1. Cancer Exosomal lncRNAs and Their Sponging miRNAs

Here, we discuss the potential connections of some exosomal lncRNAs of cancer cells to the sponging miRNAs (Table 1).

Several reported exosomal lncRNAs are summarized (Table 1). HEIH [40], LINC02418 [41], POU3F3 [40], GAPLINC [42], SNHG8 [43], SNHG16 [39], UFC1 [32], AFAP1-AS1 [39], BCAR4 [40], CCAT2 [39], CRNDE [44], DLX6-AS1 [32], HNF1A-AS1 [45], HOXA-AS2 [44], NNT-AS1 [41], PCA3 [44], PCAT1 [46], SBF2-AS1 [39], SNHG11 [47], SNHG14 [48], SNHG6 [43], SNHG7 [49], and SOX2-OT [41] are predicted to sponge several miRNAs (in cancer cells) to promote cancer proliferation. Furthermore, LNCARSR [39] and LNCRNA-ATB [39] are predicted to sponge miRNA to promote the chemoresistance and migration of cancer.

Detailed information has been provided for these exosomal lncRNAs and their sponging miRNAs (Table 1). For example, hepatocellular carcinoma upregulates the EZH2-associated lncRNA (HEIH), binds to miR-939, and then sponges the downstream function of miR-399, thus blocking the interaction between miR-939 and nuclear factor-kB (NF-κB) and improving NF-κB-mediated Bcl-xL expression for anti-apoptosis to promote tumorigenesis of colon cancer cells [50]. LINC02418, overexpressed in lung cancer tissues and cell lines, can sponge miR-4677-3p. In contrast, LINC02418 silencing inhibits the migration and proliferation of lung cancer cells, which is reversed by miR-4677-3p expression [51]. LncRNA POU class 3 homeobox 3 (POU3F3), activated by transcriptional factor SP1, improves the proliferation of cervical cancer cells by sponging miR-127-5p. The cervical cancer growth-promoting function of POU3F3 was validated by POU3F3 knockdown [52].

GAPLINC (RP11-838N2.4), overexpressed in gastric cancer, can sponge miR-211-3p to influence proliferation and angiogenesis [53] (Table 1). UFC1, upregulated in gastric cancer, is responsible for tumor growth by sponging miR-498 [56]. AFAP1 antisense RNA 1 (AFAP1-AS1) shows proliferation-promoting effects on breast cancer cells by sponging miR-195, and these effects are reversed by AFAP1-AS1 knockdown and miR-195 overexpression [57]. MiR-370-3p, downregulated in bladder cancer, was sponged by breast cancer anti-estrogen resistance 4 (BCAR4) to promote cell proliferation [58]. Colon-cancer-associated transcript 2 (CCAT2) is overexpressed in ovarian cancer cells. In comparison, CCAT2 silencing suppresses its cell proliferation, which is reversed by a miR-424 inhibitor (Table 1). Hence, CCAT2 shows sponging potential for miR-424 in the regulation of ovarian cell proliferation [59].

Colorectal neoplasia differentially expressed (CRNDE), an oncogenic lung cancer biomarker, shows proliferation-promoting effects by sponging miR-338-3p, and these effects are reversed by CRNDE knockdown [60] (Table 1). The in vivo tumor-promoting effects were validated by CRNDE knockdown. DLX6 antisense RNA 1 (DLX6-AS1), highly expressed in cervical cancer tissues and cells, showed a proliferation-promoting ability by sponging miR-199a and was reversed by the overexpression of miR-199a [61]. Similarly, DLX6-AS1 was upregulated in bladder cancer tissues showing miR-223 sponging effects and was reversed by upregulating miR-223 [62]. HNF1A antisense RNA 1 (HNF1A-AS1) was overexpressed in lung cancer and increased cell proliferation by sponging miR-17-5p [63]. HOXA cluster antisense RNA 2 (HOXA-AS2) shows high levels in leukemia cells. In comparison, HOXA-AS2 downregulation suppresses these proliferative effects. HOXA-AS2 suppresses the expression of miR-520c-3 and induces chemoresistance by sponging its function [64] (Table 1).

Liver cancer cells exhibited high levels of transhydrogenase-antisense RNA1 (NNT-AS1) and low levels of miR-363 liver cancer cells, promoting cell proliferation by sponging miR-363 [65] (Table 1). Moreover, NNT-AS1 silencing suppresses liver tumor growth. Similarly, NNT-AS1 enhances lung cancer cell proliferation by sponging miR-129-5p [66]. Prostate cancer antigen 3 (PCA3) shows a high expression in ovarian cancer and promotes cell proliferation by sponging miR-106b-5p. In contrast, the downregulation of PCA3 inhibits ovarian cell proliferation [67]. Prostate-cancer-associated ncRNA transcripts 1 (PCAT1) is upregulated in the colon [68] and ovarian [69] cancer, sponging miR-149-5p and miR-124-3p, respectively, which are responsible for cell proliferation. The proliferation-promoting functions of PCAT1 are reversed by miR-149-3p and miR-124-3p overexpression [68,69]. Additionally, PCAT1 was reported to sponge miR-3667-3p in prostate cancer cells [70]. SBF2 antisense RNA 1 (SBF2-AS1), an overexpressed marker of cervical cancer, promotes cell proliferation by sponging miR-361-5p. In contrast, the downregulation of SBF2-AS1 inhibits cell proliferation in vitro and in vivo, which is reversed by miR-361-5p inhibitors [71]. SOX2-OT, which is overexpressed in high-grade malignancy, can sponge miR-194-5p to promote the proliferation of gastric cancer cells [75] (Table 1). SOX2OT knockdown inhibits gastric tumor growth by downregulating the EMT response.

Several members of the small nucleolar RNA host gene (SNHG) family show similar proliferation-promoting functions to cancer cells [47,54,55,72,73,74] (Table 1). For example, SNHG8 and SNHG16, overexpressed biomarkers for colon cancer tissues and cells, were validated to sponge and downregulate miR-663 [54] and miR-200a-3p [55] functions, respectively, thus causing tumor growth. SNHG11 exhibits high serum levels in pancreatic cancer patients. SNHG11 promotes the proliferation of pancreatic cancer cells by sponging miR-324-3p. The downregulation of SNHG11 shows the opposite effects and recovers by suppressing miR-324-3p expression [47]. SNHG14, an oncogenic marker for lung [72] and pancreatic [73] cancer cells, promotes cell proliferation by sponging miR-340-5p and miR-101-3p, respectively. Additionally, SNHG6 and SNHG7 are highly expressed in colon cancer, leading to the proliferation of colon and breast cancer cells by sponging miR-181a-5p [74] and miR-186-5p, respectively (Table 1). SNHG6 knockdown suppresses colon tumor growth in a xenograft model [74].

Furthermore, two sponging miRNAs were shown to be responsible for chemoresistance and migration. The lncRNA regulator of Akt signaling associated with HCC and RCC (LNCARSR; lnc-TALC) overexpression was shown to be accountable for temozolomide (TMZ) resistance and associated with glioblastoma recurrence. LNCARSR can sponge miR-20b-3p to enhance c-Met expression and improve chemoresistance to TMZ [76]. LncRNA-ATB stimulated astrocytes to enhance glioma cell migration by sponging miR-204-3p [16] (Table 1).

In summary, several cancer exosomal lncRNAs sponge miRNAs and promote cancer cell proliferation.

### 2.2. The Exosomal Processing Targets of Exosomal lncRNA-Sponging miRNAs

Sponging miRNAs, such as miR-939-5p, miR-4677-3p, miR-127-5p, miR-211-3p, miR-663a, miR-200a-3p, miR-498, and miR-20b-3p, cannot retrieve the predicted targeting to exosomal processing genes (Table 1). In contrast, some proliferation-associated sponging miRNAs, such as miR-195-5p, miR-370-3p, miR-424-5p, miR-338-3p, miR-199a-5p, miR-223-3p, miR-17-5p, miR-520c-3p, miR-363-3p, miR-129-5p, miR-106b-5p, miR-149-5p, miR-124-3p, miR-3667-3p, miR-361-5p, miR-324-3p, miR-340-5p, miR-101-3p, miR-181a-5p, miR-186-5p, miR-194-5p, and miR-204-3p, are predicted to target exosomal processing genes (Table 1).

Exosomal processing genes are differentially regulated by sponging miRNAs. Some exosomal processing genes overlap in different miRNAs (Table 1). For example, exosomal processing genes such as MYO5B are targeted by the putative sponging miR-195-5p, miR-424-5p, miR-223-3p, miR-17-5p, miR-106b-5p, and miR-124-3p. RAB11A is targeted by miR-370-3p, miR-338-3p, miR-520c-3p, and miR-124-3p. ATP9A is targeted by miR-370-3p, miR-199a-5p, miR-129-5p, and miR-186-5p. VPS4A is targeted by miR-195-5p, miR-424-5p, miR-149-5p, and miR-340-5p. VPS4B is targeted by miR-363-3p, miR-129-5p, miR-124-3p, and miR-324-3p. RAB27A is targeted by miR-124-3p and miR-101-3p. PDCD6IP is targeted by miR-129-5p and miR-340-5p. Other exosomal processing genes are targeted as sponging a single miRNA (Table 1). Consequently, these exosomal processing targets for sponging miRNAs are available from the miRDB database.

## 3. Relationship between Tumor Microenvironments (TMEs), Sponging miRNAs, and Exosomal Processing Targets

### 3.1. TME and Its Associated lncRNAs

The TME contains several cell types, such as tumor cells, cancer-associated fibroblasts (CAFs), cancer stem cells (CSCs), tumor-associated macrophages (TAMs), natural killer cells, and myeloid-derived suppressor cells [77,78]. This review focuses on the lncRNAs of CAFs, CSCs, and TAMs.

CAFs are activated fibroblasts that secrete several bioactive components to enhance tumor growth, metastasis, and drug stance [79]. CSCs are a minor subpopulation of cancer cells that exhibit the self-renewal ability and can initiate tumor differentiation and development [80,81]. TAMs are macrophages that enhance the establishment of the TME [82] by upregulating immunosuppressive M2-like polarization in cancers that play a critical role in the migration and invasion of carcinogenesis [83].

Communication between those TME cells may stimulate cancer exosomes to increase carcinogenesis. Several TME-associated lncRNAs are well-understood [78]. However, the potential impacts on downstream miRNAs are rarely discussed, particularly regarding the sponging effects of TME-associated lncRNAs on miRNAs. Moreover, the putative exosomal processing targets of these predicted miRNAs have not been illustrated to date.

The potential connections of some TME-associated lncRNAs to miRNAs (Table 2) are discussed in the following. LncRNAs for some TME-associated cells, such as CAFs, CSCs, and TAMs, are included [78]. For example, some lncRNAs, such as cancer susceptibility candidate 9 (CASC9), POU3F3, small nucleolar RNA host gene 3 (SNHG3), CDKN2B-AS1 (ANRIL), colorectal cancer-associated lncRNA (CCAL), and ZEB2-AS1 (ZEB2NAT), are upregulated in CAFs [78]. HAND2 antisense RNA 1 (HAND2-AS1), lncRNA transcription factor 7 (TCF7), Lnc34a, lncRNA SMARCA2 (BRM) associated (LNCBRM), DiGeorge syndrome critical region gene 5 (DGCR5), DLX6-AS1, and LINC01567 (LOCCS) are overexpressed in CSCs [78]. LINC01089 (LIMT), TP53COR1 (linc-p21), RP11-361F15.2, ribonuclease P RNA component H1 (RPPH1) [78], FGD5 antisense RNA 1 (FGD5-AS1) [84], and lncRNA HLA complex group 18 (HCG18) [85] are upregulated in TAMs (Table 2). Consequently, these TME-associated lncRNAs can sponge several miRNAs of cancer cells.

### 3.2. Potential Functions of CAF-Associated lncRNAs That Sponge miRNAs and Modulate miRNA-Targeted Exosomal Processing Genes

In the case of CAF, several TME-associated lncRNAs were reported (Table 2). CASC9, POU3F3, SNHG3, CDKN2B-AS1, and ZEB2-AS1 are predicted to sponge several miRNAs (in cancer cells) to promote cancer proliferation. Furthermore, CCAL is predicted to sponge miRNA to promote cancer metastasis.

Detailed information is presented for these CAF-associated lncRNAs and their sponging miRNAs (Table 2). For example, CASC9 exhibits oncogenic function, and the CASC9-1 transcript is overexpressed in cervical cancer cells to increase proliferation by sponging miR-383-5p, which is reversed by CASC9 knockdown [86]. The sponging information of POU3F3 is provided in Table 1, while SNHG3, which is enriched in osteosarcoma, promotes proliferation by sponging miR-196a-5p [87]. CDKN2B-AS1, which is highly expressed in oral and ovarian cancer, promotes proliferation or suppressed apoptosis by sponging miR-125a [88] and miR-411-3p [89], respectively. ZEB2-AS1, enriched in bladder cancer cells and tissues, enhances proliferation and suppresses apoptosis by sponging miR-27b-3p, which is reversed by a miR-27b mimic [90]. Furthermore, CCAL is highly expressed in gastric cancer, and its expression levels are correlated with the metastasis stage. CCAL can sponge miR-149 and regulate metastasis of gastric cancer cells [91] (Table 2). The in vivo functions of CCAL have been validated; CCAL knockdown suppresses tumor growth and metastatic nodules in lungs in a xenograft nude mice model [91].

Some sponging miRNAs, such as miR-383-5p, miR-127-5p, and miR-196a-5p, cannot retrieve the predicted targeting to exosomal processing genes (Table 2). In contrast, some sponging miRNAs, such as miR-125a-5p, miR-411-3p, and miR-27b-3p, are predicted to target exosomal process genes such as VPS4B, SDCBP, and SMPD3, respectively. For comparison, metastasis-associated miR-149-5p is predicted to target CD34 and VPS4A (Table 2).

Consequently, CAF-associated lncRNAs sponging several miRNAs probably cause the proliferation or metastasis of cancer cells (Table 2). By data mining in respective depositories, these CAF-associated lncRNAs show the potential modulation of sponging miRNAs and targeting exosomal processing. This will be helpful in future investigations of exosome biogenesis involving these CAF-associated lncRNAs.

### 3.3. Potential Functions of CSC-Associated lncRNAs That Sponge miRNAs and Modulate miRNA-Targeted Exosomal Processing Genes

In the case of CSCs, several TME-associated lncRNAs were reported (Table 2). TCF7 is predicted to sponge miRNA (in cancer cells) to promote cancer invasion. Lnc34a, LNCBRM, DLX6-AS1, and LINC01567 are predicted to sponge several miRNAs (in cancer cells) to promote proliferation. Furthermore, HAND2-AS1 and DGCR5 are predicted to sponge miRNA to inhibit the proliferation and radiosensitivity of cancer, respectively.

Detailed information has been provided for these CSC-associated lncRNAs and their sponging miRNAs (Table 2). For example, TCF7 promotes the invasion of cervical cancer cells by sponging miR-155-5p, which is reversed by TCF7 downregulation [92]. Moreover, TCF7 knockdown suppresses cervical tumor growth. Lnc34a shows high levels in colon CSCs, causes an asymmetric division of CSCs, and improves colon cancer proliferation by sponging miR-34a [93]. LNCBRM overexpression enhances colon cancer cell proliferation and invasion by sponging miR-204-3p, which is reversed by LNCBRM knockdown [94]. The sponging information of DLX6-AS1 is provided in Table 1. WLINC01567 was overexpressed in colon CSCs to increase proliferation by sponging miR-93-mediated tumor suppression, which was reversed by LINC01567 knockdown [95] (Table 2).

In contrast, HAND2-AS1, which is downregulated in chronic myeloid leukemia, inhibits proliferation by targeting and sponging miR-1275 [96] (Table 2). DGCR5 promotes radioresistance and CSC proliferation for laryngeal cancer cells by sponging miR-195 [97] and miR-506 [98], respectively. DGCR5 knockdown shows the opposite effect. The tumor-promoting effects of DGCR5 in laryngeal cancer cells were validated by downregulating DGCR5 [97].

Some sponging miRNAs, such as miR-155-5p and miR-1275, cannot retrieve the predicted targeting to exosomal process genes (Table 2). In contrast, some sponging miRNAs, such as miR-34a-5p, miR-204-3p, miR-199a-5p, miR-223-3p, and miR-93-5p, are predicted to target exosomal process genes such as VPS4A, RAB11A, ATP9A, and MYO5B, respectively. In comparison, metastasis-associated miR-195-5p and miR-506-3p are predicted to target MYO5B, VPS4A, MYO5B, RAB11A, RAB27A, SDC1, SDC4, STEAP3, and VPS4B (Table 2).

Consequently, CSC-associated lncRNAs sponge several miRNAs, which may promote or inhibit proliferation and enhance the invasion and radioresistance of cancer cells (Table 2). Using data mining, these CSC-associated lncRNAs modulate sponging miRNA-targeting exosomal processing. This will be helpful for future investigations of exosome biogenesis involving these CSC-associated lncRNAs.

### 3.4. Potential Functions of TAM-Associated lncRNAs That Sponge miRNAs and Modulate the miRNA-Targeted Exosomal Process

In the case of TAMs, several TME-associated lncRNAs have been reported (Table 2). RP11-361F15.2 and RPPH1 are predicted to sponge miRNA (in cancer cells) to promote cancer invasion. FGD5-AS1 and HCG18 are predicted to sponge several miRNAs of cancer cells to promote cancer proliferation. Furthermore, LINC01089 and TP53COR1 are predicted to sponge miRNA to inhibit the proliferation of cancer.

Detailed information is provided for these TAM-associated lncRNAs and their sponging miRNAs (Table 2). For example, RP11-361F15.2 shows more significant upregulation in osteosarcoma than the normal control, contributing to invasion by promoting the M2-like polarization of TAM, which is reversed by RP11-361F15.2 downregulation. RP11-361F15.2 sponges and downregulates miR-30c-5p expression, contributing to the promotion of invasion in osteosarcoma [83]. Moreover, overexpression of RP11-361F15.2 enhances the growth of xenograft osteosarcoma [83]. RPPH1 shows more unique expression in lung cancer than normal cells, contributing to cisplatin resistance (Table 2). RPPH1 downregulation suppresses the invasive ability of lung cancer cells, while RPPH1 overexpression shows the opposite effects by sponging miR-326 expression [99]. FGD5-AS1, which shows high expression in glioblastoma cells, is essential for cancer progression. This was reversed by FGD5-AS1 knockdown, leading to binding of miR-129-5p and suppression of miR-129-5p expression [100]. Similarly, FGD5-AS1 shows high expression in colon cancer, enhancing proliferation and inhibiting apoptosis, which is reversed by miR-302e knockdown. MiR-302e is bound to FGD5-AS1. Collectively, FGD5-AS1 upregulated the sponging effects on miR-129-5p [100] and miR-302e [101] and promoted the progression of colon cancer and glioblastoma, respectively. HCG18 was overexpressed in colon cancer for increased proliferation that was reversed by HCG18 knockdown, which induced miR-1271-5p overexpression. HCG18 levels were proportional to the degree of colon cancer malignancy, and this relationship is thought to be due to sponging of miR-1271-5p [102] (Table 2).

Furthermore, LINC01089, showing low levels in cervical cancer, was reversely correlated with tumor growth and lymph node metastasis as a consequence of sponging miR-27a-3p, and this was reversed by LINC01089 knockdown [103] (Table 2). TP53COR1 inhibited liver cancer cell proliferation by sponging and downregulating miR-9 expression [104].

Sponging miRNAs, such as miR-30c-5p, cannot retrieve the predicted targeting to exosomal processing genes (Table 2). In contrast, some invasion-associated sponging miRNAs, such as miR-30c-5p and miR-326, were predicted to target exosomal processing genes such as RAB11A and CD34, respectively. Proliferation-associated miR-129-5p was predicted to target ATP9A, PDCD6IP, and VPS4B, while miR-302e targets RAB11A, RAB7A, SDC1, and SMPD3. Moreover, antiproliferation-associated miR-1271-5p targets MYO5B and RAB7A. MiR-9-5p targets CD34, PDCD6IP, SDC1, SMPD3, and STEAP3 (Table 2).

Consequently, TAM-associated lncRNAs sponge several miRNAs and may promote or inhibit proliferation and enhance the invasion of cancer cells (Table 2). Using data mining, these TAM-associated lncRNAs show the potential modulation of the sponging miRNA-targeted exosomal processing. This will be helpful in the future investigation of exosome biogenesis involving these TAM-associated lncRNAs.

## 4. The Potential Sponging miRNAs and Exosomal Processing Targets for Natural-Product-Modulated lncRNAs

Several lncRNA-modulating natural products have been reported. However, the potential response of exosomal processing genes in the modulation of the lncRNA–miRNA axis of natural products remains unclear.

A panel of natural-product-downregulated (Section 4.1) and -upregulated (Section 4.2) exosomal lncRNAs that sponge LncTarD [34]-predicted miRNAs are summarized (Table 3). Subsequently, these sponging miRNAs may target miRDB [38]-predicted exosomal processing genes (Section 4.3). An overview of the natural-product-affected lncRNA–miRNA–exosomal processing axis is provided (Section 4.4). Notably, the connection between the lncRNA–miRNA–exosomal processing axis and natural products warrants a thoughtful assessment in the future.

### 4.1. The Predicted Sponging miRNAs of Natural-Product-Downregulated lncRNAs

The potential connections between some natural-product-downregulated lncRNAs and sponging miRNAs are shown (Table 3).

Anacardic acid [105] (Table 3) downregulates MIR210HG, which is upregulated in breast cancer. Moreover, MIR210HG promotes the invasion of breast cancer cells by sponging miR-1226-3p [106]. The in vitro and in vivo invasion and metastasis of breast cancer cells were inhibited by silencing MiR210HG. Consequently, anacardic acid may suppress cancer cell invasion by regulating the MIR210HG–miR-1226-3p axis. Anisomycin [78,107] (Table 3) downregulates BACE1-antisense RNA (BACE1-AS), which is upregulated in liver cancer cells. Moreover, BACE1-AS promotes the invasion of liver cancer cells by sponging miR-377-3p [108]. Hence, anisomycin shows the potential for the anti-invasion of liver cancer cells by modulating the BACE1-AS–miR-377-3p axis.

β-elemene [78,109] (Table 3) downregulates highly upregulated liver cancer (HULC), which is upregulated in osteosarcoma and pancreatic cancer cells. Moreover, a xenograft model has validated the in vivo lymphoma-suppressive effects of β-elemene [109]. HULC promotes the cell invasion of osteosarcoma and pancreatic cancer cells by sponging miR-122-5p [110] and miR-133b [111], respectively. In contrast, HULC knockdown inhibits osteosarcoma and pancreatic cancer cell invasion by upregulating miR-122-5p [110] and miR-133b [111], respectively. β-elemene shows the potential for anti-invasion of these cancer cells by modulating the HULC–miR-122-5p/miR-133b axis. Polydatin [112] and solamargine [113] (Table 3) downregulate taurine-upregulated 1 (TUG1), which shows high expression in nasopharyngeal cancer cells. TUG1 promotes cell proliferation and migration accompanied by the low expression of miR-384. TUG1 knockdown inhibits nasopharyngeal cancer progression and epithelial–mesenchymal transition (EMT) by upregulating miR-384. These results suggest that TUG1 promotes the migration of nasopharyngeal cancer cells by sponging miR-384 [114]. Moreover, TUG1 knockdown suppresses tumor growth of the nasopharyngeal cancer cell xenograft model by upregulating miR-384 and downregulating EMT progression [114]. Therefore, polydatin and solamargine may inhibit the migration of nasopharyngeal cancer cells by regulating the TUG1–miR-384 axis. Additionally, curcumin [115] (Table 3) downregulates LINC-RoR, where the LINC-RoR levels are correlated with the pluripotent state of endometrial CSCs. The differentiation of CSCs upregulates miR-145 expression. The upregulation of miR-145 suppresses LINC-RoR expression and colony formation [116]. These results suggest that LINC-RoR can sponge miR-145 to regulate endometrial CSCs [116] (Table 3). Therefore, curcumin may inhibit the differentiation of endometrial CSCs by modulating the LINC-RoR–miR-145 axis.

In addition to invasion, migration, and differential modulating effects, the function of the remaining natural-product-downregulated lncRNAs is promotion of the proliferation of cancer cells (Table 3). For example, curcumin [117], sulforaphane [118], bharangin [105], ginsenosides [105], and triptolide [119] downregulate H12. In gastric cancer patients, H12 is upregulated, and miR-141-3p is downregulated [120]. Similarly, H19 is upregulated in glioma cells [122]. H19 increases the proliferation of gastric cancer and glioma cells by sponging miR-141 [120], miR 22-3p [121], and miR-152 expression [122] (Table 3). Moreover, H19 knockdown suppresses glioma growth in a xenograft nude mouse model [122]. Therefore, curcumin, sulforaphane, bharangin, ginsenosides, and triptolide may inhibit cancer proliferation by regulating the H19–miR-141/miR 22-3p/miR-152 axis.

Moreover, sulforaphane [105] (Table 3) downregulates LINC01116, which directly binds to miR-592 and sponges miR-592 expression to promote acute myeloid leukemia proliferation in vivo [123]. The LINC01116–miR-592 axis may play a critical role in regulating proliferation. Solamargine [113] downregulates HOXA distal transcript antisense RNA (HOTTIP), which is upregulated in prostate cancer cells to promote proliferation by sponging miR-216a-5p [124]. Therefore, solamargine suppresses cancer cell proliferation by modulating the HOTTIP–miR-216a-5p axis. Additionally, formononetin [57] (Table 3) downregulates AFAP1-AS1, where AFAP1-AS1 overexpression sponges miR-195-5p to promote breast cancer cell proliferation [57]. Consequently, formononetin may inhibit cancer cell proliferation by modulating the AFAP1-AS1–miR-195-5p axis.

Resveratrol [105] (Table 3) downregulates MIR4435-2HG, which is upregulated in melanoma to stimulate proliferation by sponging miR-802, which is reversed by downregulating MIR4435-2HG [125]. Therefore, resveratrol inhibits the proliferation of melanoma by modulating the MIR4435-2HG–miR-802 axis. Luteolin [105] (Table 3) downregulates BRAF-activated noncoding RNA (BANCR), which is upregulated in pancreatic cancer tissues. BANCR enhances proliferation by sponging miR-195-5p, which is reversed by BANCR downregulation [126]. Therefore, luteolin may inhibit pancreatic cancer cell proliferation by modulating the BANCR-2HG–miR-195-5p axis.

Hyperoside and baicalein [78,127] (Table 3) downregulate colon-cancer-associated transcript 1 (CCAT1), which is overexpressed in glioma and lung cancer tissues. CCAT1 increases proliferation by sponging miR-410-3p [128] and miR-152-5p [129]. Hyperoside and baicalein may suppress glioma and lung cancer cell proliferation by regulating the CCAT1–miR-410-3p/miR-152-5p axis. Similarly, CCAT2, which is enriched in ovarian cancer tissues and cells, promotes proliferation by sponging miR-424-5p [59]. Gemini curcumin [130] shows suppressing effects on CCAT2, leading to the inhibition of ovarian cancer cell proliferation via the CCAT2–miR-424-5p axis. Calycosin [105] (Table 3) downregulates Ewing-sarcoma-associated transcript 1 (EWSAT1), which enhances the proliferation of cervical and nasopharyngeal cancer cells by sponging miR-330-5p [131,132]. Consequently, calycosin may exert antiproliferative effects on these cancer cells by modulating the EWSAT1–miR-330-5p axis.

Different natural products may target the same lncRNA. Berberis, calycosin, curcumin, 3,30-diindolylmethane, genistein, and silibinin [105] (Table 3) show downregulating effects on HOX transcript antisense RNA (HOTAIR). In liver cancer tissues and cells, HOTAIR is overexpressed, and miR-217-5p is downregulated. HOTAIR promotes liver cancer cell proliferation by sponging miR-217-5p, which is reversed by HOTAIR knockdown [133]. The role of in vivo liver cancer progression was also validated by HOTAIR knockdown. Therefore, these drugs [105] may suppress liver cell proliferation by modulating the HOTAIR–miR-217-5p axis. Melatonin [134] (Table 3) downregulates the lncRNA just proximal to XIST (JPX), which is overexpressed in lung cancer tissues. JPX enhances the proliferation of lung cancer cells by sponging miR-362-3p [135]. Hence, melatonin has antiproliferation potential for lung cancer by regulating the JPX–miR-362-3p axis.

Epigallocatechin-3-gallate [105] (Table 3) downregulates LINC00511, which is upregulated in gastric and liver cancer tissues and cells. LINC00511 promotes the proliferation of gastric and liver cancer cells by sponging miR-515-5p [136] and miR-424 [137], respectively. In contrast, LINC00511 knockdown suppresses the proliferation of breast [136] and liver [137] cancer cells. Accordingly, epigallocatechin-3-gallate may suppress breast and liver cancer cells by regulating the LINC00511–miR-515-5p/miR-424 axis. Betulinic acid [78], quercetin [138], and resveratrol K [105] (Table 3) downregulate metastasis-associated lung adenocarcinoma transcript 1 (MALAT1). Colon and breast cancer overexpress MALAT1 and stimulate proliferation by sponging miR-101-3p [139] and miR-129-5p [139], respectively. Consequently, these drugs [78,105,138] may inhibit the proliferation of colon and breast cancer cells by modulating the MALAT1–miR-101-3p/miR-129-5p axis.

Anisomycin [78,141,142] (Table 3) downregulates maternally expressed gene 3 (MEG3), which exhibits low levels in oral and colon cancer tissues and cells to promote proliferation by sponging miR-21-5p [143] and miR-708-5p [144], respectively. MiR-708-5p promotes colon tumor growth in Apc(min) mice by downregulating MEG3 [144]. Hence, anisomycin may inhibit oral and colon cancer cell proliferation by regulating the MEG3–miR-21-5p/miR-708-5p axis. Quercetin [138] (Table 3) downregulates myocardial-infarction-associated transcript (MIAT). Acute myeloid leukemia and ovarian cancer upregulate MIAT, increasing the proliferation by sponging miR-495 [145] and miR-330-5p [146], respectively. Moreover, MIAT knockdown suppresses the progression of acute myeloid leukemia in immunodeficient NOD-SCID mice [145]. Accordingly, quercetin may exert antiproliferative effects on leukemia and ovarian cancer cells by modulating the MIAT–miR-495/miR-330-5p axis.

The inhibition of prostate-specific transcript 1 (PCGEM1) upregulates miR-145 expression, inhibiting the proliferation and xenograft tumor growth of prostate cancer cells. This suggests that PCGEM1 promotes prostate cancer cell proliferation by sponging miR-145 [147]. 3,30-Diindolylmethane [105] (Table 3) downregulates PCGEM1. Accordingly, 3,30-dindolylmethane may suppress the proliferation of prostate cancer cells by regulating the PCGEM1–miR-145 axis. Curcumin [78] and cardamonin [148] downregulate Pvt1 oncogene (PVT1). Gallbladder cancer tissues and cells upregulate PVT1 and downregulate miR-30d-5p, thus promoting proliferation by sponging miR-30d-5p expression, which is reversed by PVT1 knockdown [149]. Therefore, curcumin and cardamonin can potentially suppress the proliferation of gallbladder cancer cells by modulating the PVT1–miR-30d-5p axis. Epigallocatechin-3-gallate [105] (Table 3) also downregulates SOX2OT. Additionally, SOX2OT sponges miR-194-5p, which promotes gastric cancer cell proliferation [75]. Accordingly, epigallocatechin-3-gallate may exert antiproliferative effects on gastric cancer cells by regulating the SOX2OT–miR-194-5p axis.

Gambogic acid [105] (Table 3) downregulates sprouty4-Intron 1 (SPRY4-IT1), which is upregulated in colon cancer cells and enhances proliferation by sponging and downregulating miR-101-3p expression [150]. Gambogic acid may suppress the proliferation of colon cancer cells by regulating the SPRY4-IT1–miR-101-3p axis. Huaier [78,151] downregulates P73 antisense RNA 1 (TP73-AS1). Cervical cancer tissues and cells overexpress TP73-AS1 and downregulate miR-329-3p to promote proliferation. Mechanistically, TP73-AS1 sponges miR-329-3p and suppresses its function [152]. Therefore, Huaier may induce antiproliferative effects on cervical cancer cells by regulating the TP73-AS1–miR-329-3p axis [152]. Curcumin [105] and usnic acid [153] (Table 3) downregulate urothelial carcinoma-associated 1 (UCA1), which is upregulated in gastric and lung cancer and promotes proliferation by sponging miR-26a-5p [154] and miR-144-3p [155], respectively. Moreover, UCA1 knockdown inhibits tumor growth in a gastric cancer cell xenograft model by triggering apoptosis [155]. Consequently, the modulation of the UCA1–miR-26a-5p/miR-144-3p axis may play a crucial role in inhibiting the proliferation of gastric and lung cancer cells.

Atractylenolide II [156] and platycodin D [157] (Table 3) downregulate the X-inactive-specific transcript (XIST). Moreover, platycodin D inhibits bladder tumor growth by downregulating LncRNA-XIST [157]. Liver cancer cells show high levels of XIST-enhancing proliferation by sponging miR-200b-3p [158]. Knockdown by sh-XIST suppresses tumor formation in a xenograft model [158]. The antiproliferative effects of atractylenolide II and platycodin D are possibly mediated by modulating the XIST–miR-200b-3p axis. Silibinin [105] (Table 3) downregulates ZNFX1 antisense RNA 1 (ZFAS1). Nasopharyngeal cancer cells contain high levels of ZNFX1 antisense RNA 1 ZFAS1 and low levels of miR-135a-5p. ZFAS1 knockdown suppresses nasopharyngeal cancer cell proliferation by sponging miR-135a-5p [159]. Therefore, silibinin may inhibit the proliferation of nasopharyngeal cancer cells by regulating the ZFAS1–miR-135a-5p axis.

Consequently, natural products downregulate some lncRNAs and fail to sponge miRNAs which are responsible for promoting the proliferation, migration, and invasion of cancer cells. To conclude, natural products suppress the miRNA-sponging effects of lncRNAs, thus leading to the inhibition of proliferation, migration, and invasion.

### 4.2. Predicted Sponging miRNAs of lncRNAs Upregulated by Natural Products

The potential connections of some natural-product-upregulated lncRNAs to the sponging of miRNAs are reviewed here (Table 3).

Curcumin [105] (Table 3) upregulates tumor-suppressor candidate 7 (TUSC7), which exhibits low levels in esophageal cancer and glioblastoma tissues and cells, providing the inhibition of chemoresistance. TUSC7 inhibits the chemoresistance of esophageal cancer and glioblastoma cells by sponging miR-224-5p [160] and miR-10a-5p [161], respectively. Moreover, overexpression of TUSC7 inhibits tumor growth and chemoresistance in the esophageal cancer xenograft model [160]. Consequently, curcumin may suppress the chemoresistance of esophageal cancer and glioblastoma cells by modulating the TUSC7–miR-224-5p/miR-10a-5p axis.

Baicalein [162] (Table 3) upregulates PAX8-AS1 (PAX8 antisense RNA 1), which is downregulated in papillary thyroid cancer tissues. PAX8-AS1-N knockdown enhances tumor growth in a breast xenograft model [162]. PAX8-AS1 overexpression suppresses the proliferation of thyroid cancer cells by binding and sponging miR-96-5p [163]. The PAX8-AS1–miR-96-5p axis may participate in baicalein-induced antiproliferative effects on thyroid cancer cells. Bharangin, curcumin, gambogic acid [105], and corylin [164] upregulate growth-arrest-specific 5 (GAS5) levels of B lymphocytic leukemia, gastric cancer, and breast cancer cells. GAS5 shows negative regulation of proliferation by sponging miR-222-3p [165], miR-222-3p [166], and miR-196a-5p [167]. Therefore, the drugs [105,164] may exert antiproliferative effects on leukemia, gastric cancer, and breast cancer cells by modulating the GAS5–miR-222-3p/miR-222-3p/miR-196a-5p axis. Ginsenosides [105] (Table 3) upregulate STXBP5 antisense RNA 1 (STXBP5-AS1). Cervical cancer cells show low levels of STXBP5-AS1 and high levels of miR-96-5p. In contrast, STXBP5-AS1 overexpression inhibits cervical cancer cell proliferation by sponging miR-96-5p expression [168]. The STXBP5-AS1–miR-96-5p axis may be crucial to the ginsenoside-induced antiproliferative effects on cervical cancer cells. Furthermore, resveratrol [105] upregulates prostate-cancer-associated transcript 29 (PCAT29), which downregulated in lung cancer to stimulate proliferation. In contrast, PCAT29 overexpression inhibits lung cancer cell proliferation by sponging miR-494 [169] (Table 3). Accordingly, resveratrol may induce antiproliferative effects on lung cancer cells by modulating the PCAT29–miR-494 axis.

Consequently, natural products upregulate some lncRNAs and sponge miRNAs which are responsible for inhibiting proliferation and chemoresistance. Collectively, natural products promote the miRNA-sponging effects of lncRNAs, thus leading to the inhibition of proliferation and chemoresistance.

### 4.3. The Predicted Exosomal Processing Targets of Sponging miRNAs for Natural-Product-Downregulated and -Upregulated lncRNAs

For natural-product-downregulated and -upregulated lncRNAs, their sponging miRNAs, such as miR-1226-3p, miR-377-3p, miR-122-5p, miR-133b, miR-145, miR-141-3p, miR-22-3p, miR-152, miR-592, miR-216a-5p, miR-222-3p, miR-196a-5p, and miR-494, cannot retrieve the predicted targeting to exosomal process genes (Table 3). In contrast, the migration-associated sponging miRNA, miR-384, is predicted to target the exosomal process gene PDCD6IP. The chemoresistance-associated sponging miRNA, miR-96-5p, is predicted to target MYO5B, RAB27A, and RAB7A. Most sponging miRNAs are responsible for promoting proliferation (Table 3).

Different sponging miRNAs can regulate the same exosomal processing genes (Table 3). For example, the exosomal processing gene PDCD6IP is targeted by miR-330-5p, miR-217-5p, miR-362-3p, and miR-135a-5p. MYO5B is targeted by the putative sponging miR-195-5p, miR-424-5p, miR-21-5p, and miR-9b-5p. RAB11A is targeted by miR-410-3p, miR-515-5p, miR-21-5p, miR-30d-5p, and miR-26a-5p. RAB27A is targeted by miR-101-3p and miR-96-5p. RAB7A is targeted by miR-802 and miR-96-5p. STAM is targeted by miR-145-5p and miR-200b-3p. VP4SA is targeted by miR-195-5p, miR-424-5p, and miR-515-5p. VPS4B is targeted by miR-129-5p and miR-26a-5p. ATP9A is targeted by miR-217-5p, miR-129-5p, and miR-224-5p. SDC1 is targeted by miR-152-5p, miR-708-5p, and miR-10a-5p. SDC4 is targeted by miR-802 and miR-194-5p. SMPD3 is targeted by miR-152-5p, miR-144-3p, and miR-10a-5p. Furthermore, the remaining exosomal processing genes, such as SDCBP, CD34, STEAP3, and PRKN, are targeted by miR-10a-5p, miR-330-5p, miR-217-5p, and miR-200b-3p (Table 3), respectively. In conclusion, these exosomal processing targets for natural-product-modulated, lncRNA-sponging miRNAs became available by mining from the miRDB database.

### 4.4. Overview of Natural Products That Modulate the Exosomal lncRNA–miRNA Axis to Regulate Exosomal Processing

The relationship between natural products, their exosomal lncRNA-modulating effects, and their sponging miRNAs and potential targets of exosomal processing are summarized (Table 3). To concentrate on the final step of exosomal processing targets, exosomal-process-centric relationships are represented (Table 4).

In general, different sponging miRNAs can target the same exosomal processing gene (Table 4). For example, ATP9A is targeted by miR-217-5p, miR-129-5p, and miR-224-5p. MYO5B is targeted by miR-195-5p, miR-424-5p, miR-21-5p, and miR-96-5p. RAB11A is targeted by miR-410-3p, miR-515-5p, miR-21-5p, miR-30d-5p, and miR-920a-5p. RAB11A is targeted by miR-410-3p, miR-515-5p, miR-21-5p, miR-30d-5p, and miR-26a-5p. RAB27A is targeted by miR-101-3p, miR-96-5p, miR-21-5p, miR-30d-5p, and miR-26a-5p. RAB7A is targeted by miR-802 and miR-96-5p. PDCD6IP is targeted by miR-330-5p, miR-217-5p, miR-362-3p, miR-129-5p, miR-330-5p, miR-329-3p, miR-384, miR-144-3p, and miR-135a-5p. SDC1 is targeted by miR-152-5p, miR-708-5p, and miR-10a-5p. SDC4 is targeted by miR-802, miR-495-3p, and miR-194-5p. SMPD3 is targeted by miR-152-5p, miR-10a-5p, and miR-144-3p. STAM is targeted by miR-128-3p, miR-145-5p, and miR-200b-3p. SDC1 is targeted by miR-152-5p, miR-708-5p, and miR-10a-5p. VPS4A is targeted by miR-195-5p and miR-424-5p. VPS4B is targeted by miR-128-3p, miR-129-5p, and miR-144-3p (Table 4).

Moreover, the same miRNAs can be sponged by different lncRNAs. For example, miR-101-3p is targeted by MALAT1 and SPRY4-IT1 (Table 4). MiR-330-5p is targeted by EWSAT1 and MIAT. Similarly, the same lncRNAs can sponge different lncRNAs. CCAT1 sponges miR-410-3p and miR-152-5p; LINC00511 sponges miR-424-5p and miR-155-5p; MALAT1 sponges miR-129-5p, miR-101-3p, and miR-129-5p; MEG3 sponges miR-21-5p and miR-708-5p; MIAT sponges miR-330-5p and miR-495-5p; TUSC7 sponges miR-224-5p and miR-10a-5p; and UCA1 sponges miR-26a-5p and miR-144-3p (Table 4).

Consequently, the relationships between natural products, lncRNAs, sponging miRNAs, and exosomal processing targets are clearly explored.

## 5. Conclusions

Cancerous exosomes contain complicated biomolecule compositions that promote cancer progression. Among other issues, this review focused on exploring the relationship between lncRNAs, miRNAs, and exosomal processing targets. Generally, the lncRNA–miRNA–target axis is straightforward in terms of signaling. However, lncRNAs and miRNAs regulate many downstream effectors, and all complicated signaling regulations are arranged in a cascade. To clarify this rationale, only the sponging miRNAs of exosomal lncRNAs were discussed.

Moreover, several natural products that modulate the exosomal lncRNAs were described. Their potential role in sponging miRNAs and exosomal processes lacks systemic organization in the order of the lncRNA–miRNA–target axis. This gap was filled by the bioinformatic database. The potential exosomal processing targets for the sponging miRNAs were then retrieved. These sponging miRNAs and exosomal processing targets were summarized using lncTarD and miRDB databases. Consequently, the exosomal lncRNAs and their potential roles in regulating sponging miRNAs and exosomal processing were clarified. Notably, these sponging miRNAs of lncRNAs were bioinformatically predicted, although miRNA-sponging functions were derived from the literature reports with citations. Moreover, these potential candidates may belong to different cancer cell lines and exhibit tissue-specific expression. This warrants a careful investigation by wet experiments in the future. Notably, most studies in this review focus on exploring the lncRNA sponging effects on miRNAs and the targeting effects of miRNA on exosomal processing in cell models, although some of them have evidence from animal studies. A thoughtful in vivo assessment is still required to explore the roles of exosomal lncRNAs, sponging miRNAs, and exosomal processing targets in natural product experiments by animal studies.

**Figure 3:**
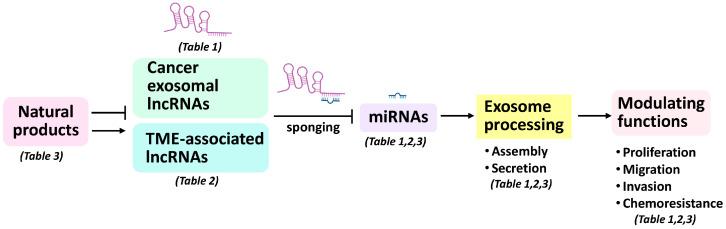
Schematic summary of the natural products acting on the exosomal lncRNA–sponging miRNA–exosomal process axis. Natural products may modulate (inhibit (T) or enhance (arrow)) cancer and TME-associated lnRNAs. Next, the lncRNAs may sponge miRNAs, and, in turn, miRNAs can modulate target exosome processing genes, providing modulating functions to cancer cells. More detailed information for each step has been mentioned in the Table 1, Table 2 and Table 3 above.

Many exosomal lncRNAs control exosomal processing changes. This review provides a future investigation direction, as encouraged by the bioinformatical prediction of several sponging miRNAs. Similarly, TME-associated and natural-product-modulated lncRNAs show similar problems that ignore the potential involvement of exosomal processing in the literature and can be solved using bioinformatic strategies. Accordingly, this review sheds light on explorations of the exosomal lncRNA–sponging miRNA–exosomal process axis and the potential impact of natural products on this axis (Figure 3), providing a possible future direction for cancer therapies using exosomal lncRNA.

## Figures and Tables

**Table 1:** The predicted sponging miRNAs of exosomal lncRNAs and predicted exosomal processing targets of these miRNAs.

Exosomal lncRNAs	Sponging miRNAs	Sponging Function	Exosomal Processing Targets
HEIH [40]	miR-939-5p (colon ca) [50]	proliferation (+)	X
LINC02418 [41]	miR-4677-3p (lung ca) [51]	proliferation (+)	X
POU3F3 [40]	miR-127-5p (cervical ca) [52]	proliferation (+)	X
GAPLINC [42]	miR-211-3p (gastric ca) [53]	proliferation (+)	X
SNHG8 [43]	miR-663a (colon ca) [54]	proliferation (+)	X
SNHG16 [39]	miR-200a-3p (colon ca) [55]	proliferation (+)	X
UFC1 [32]	miR-498 (gastric ca) [56]	proliferation (+)	X
AFAP1-AS1 [39]	miR-195-5p (breast ca) [57]	proliferation (+)	MYO5B, VPS4A
BCAR4 [40]	miR-370-3p (bladder ca) [58]	proliferation (+)	ATP9A, RAB11A, RAB7A
CCAT2 [39]	miR-424-5p (ovarian ca) [59]	proliferation (+)	MYO5B, VPS4A
CRNDE [44]	miR-338-3p (lung ca) [60]	proliferation (+)	RAB11A
DLX6-AS1 [32]	miR-199a-5p (cervical ca) [61]/miR-223-3p (bladder ca) [62]	proliferation (+)	ATP9A/MYO5B
HNF1A-AS1 [45]	miR-17-5p (lung ca) [63]	proliferation (+)	MYO5B
HOXA-AS2 [44]	miR-520c-3p (breast ca) [64]	proliferation (+)	RAB11A, SDC1
NNT-AS1 [41]	miR-363-3p (gastric ca) [65]/miR-129-5p (lung ca) [66]	proliferation (+)	VPS4B/ATP9A, PDCD6IP, VPS4B
PCA3 [44]	miR-106b-5p (ovarian ca) [67]	proliferation (+)	MYO5B, TSG101
PCAT1 [46]	miR-149-5p (colon ca) [68]/miR-124-3p (ovarian ca) [69]/miR-3667-3p (prostate ca) [70]	proliferation (+)	CD34, VPS4A/MYO5B, RAB11A, RAB27A, SDC1, VPS4B/SMPD3
SBF2-AS1 [39]	miR-361-5p (cervical ca) [71]	proliferation (+)	SDCBP
SNHG11 [47]	miR-324-3p (pancreatic ca) [47]	proliferation (+)	RAB7B
SNHG14 [48]	miR-340-5p (lung ca) [72]/miR-101-3p (pancreatic ca) [73]	proliferation (+)	PDCD6IP, VPS4A/RAB27A
SNHG6 [43]	miR-181a-5p (colon ca) [74]	proliferation (+)	PRKN
SNHG7 [49]	miR-186-5p (breast ca) [74]	proliferation (+)	ATP9A, VPS4B
SOX2-OT [41]	miR-194-5p (gastric ca) [75]	proliferation (+)	SDC4
LNCARSR [39]	miR-20b-3p (glioblastoma) [76]	chemoresistance (+)	X
LNCRNA-ATB [39]	miR-204-3p (glioma) [16]	migration (+)	RAB11A

The predicted sponging miRNAs of lncRNAs were retrieved from the lncTarD 2.0 database. The predicted exosomal processing gene targets of these sponging miRNAs were retrieved from the miRDB database (retrieval date: 1 March 2023); ca indicates cancer, and X indicates that no potential target is available from the miRDB database.

**Table 2:** Predicted sponging miRNAs of TME-associated lncRNAs and sponging miRNAs’ exosomal processing targets.

LncRNAs	LncRNA Status (TME)	Sponging miRNAs	Sponging Function	Exosomal Processing Targets
CASC9 [78]	↑CAFs	miR-383-5p (cervical ca) [86]	↑proliferation	X
POU3F3 [78]	↑CAFs	miR-127-5p (cervical ca) [52]	↑proliferation	X
SNHG3 [78]	↑CAFs	miR-196a-5p (osteosarcoma) [87]	↑proliferation	X
CDKN2B-AS1 [78]	↑CAFs	miR-125a-5p (oral ca) [88]/miR-411-3p (ovarian ca) [89]	↑proliferation	VPS4B/SDCBP
ZEB2-AS1 [78]	↑CAFs	miR-27b-3p (bladder ca) [90]	↑proliferation	SMPD3
CCAL [78]	↑CAFs	miR-149-5p (gastric ca) [91]	↑metastasis	CD34, VPS4A
TCF7 [78]	↑CSCs	miR-155-5p (cervical ca) [92]	↑invasion	X
Lnc34a [78]	↑CSCs	miR-34a-5p (colon ca) [93]	↑proliferation	VPS4A
LNCBRM [78]	↑CSCs	miR-204-3p (colon ca) [94]	↑proliferation	RAB11A
DLX6-AS1 [78]	↑CSCs	miR-199a-5p (cervical ca) [61]/miR-223-3p (bladder ca) [62]	↑proliferation	ATP9A/MYO5B
LINC01567 [78]	↑CSCs	miR-93-5p (colon ca) [95]	↑proliferation	MYO5B
HAND2-AS1 [78]	↑CSCs	miR-1275 (leukemia) [96]	↓proliferation	X
DGCR5 [78]	↑CSCs	miR-195-5p (laryngeal ca) [97]/miR-506-3p (laryngeal ca) [98]	↓radiosensitivity	MYO5B, VPS4A/MYO5B, RAB11A, RAB27A, SDC1, SDC4, STEAP3, VPS4B
RP11-361F15.2 [78]	↑TAMs	miR-30c-5p (osteosarcoma) [83]	↑invasion	RAB11A
RPPH1 [78]	↑TAMs	miR-326 (lung ca) [99]	↑invasion	CD34
FGD5-AS1 [84]	↑TAMs	miR-129-5p (glioblastoma) [100]/miR-302e (colon ca) [101]	↑proliferation	ATP9A, PDCD6IP, VPS4B/RAB11A, RAB7A, SDC1, SMPD3
HCG18 [85]	↑TAMs	miR-1271-5p (colon ca) [102]	↑proliferation	MYO5B, RAB7A
LINC01089 [78]	↑TAMs	miR-27a-3p (cervical ca) [103]	↓proliferation	X
TP53COR1 [78]	↑TAMs	miR-9-5p (liver ca) [104]	↓proliferation	CD34, PDCD6IP, SDC1, SMPD3, STEAP3

↑, enhance; ↓, inhibit. Cancer (ca); tumor microenvironment (TME); cancer-associated fibroblasts (CAFs); cancer stem cells (CSCs); tumor-associated macrophages (TAMs). The predicted sponging miRNAs of lncRNAs were retrieved from the lncTarD 2.0 database. The predicted exosomal processing gene targets of these sponging miRNAs were retrieved from the miRDB database (retrieval date: 1 March 2023). X indicates that no potential target is available in the miRDB database.

**Table 3:** Predicted sponging miRNAs and exosomal processing targets for natural-product-modulated lncRNAs.

	Natural Products	lncRNAs	Sponging miRNAs	Sponging Function	Exosomal Processing Targets
**Downregulation**	Anacardic acid [105]	MIR210HG	miR-1226-3p (breast ca) [106]	↑invasion	X
	Anisomycin [78,107]	BACE1-AS	miR-377-3p (liver ca) [108]	↑invasion	X
	β-Elemene [78,109]	HULC	miR-122-5p (osteosarcoma) [110]/miR-133b (pancreatic ca) [111]	↑invasion	X
	Polydatin [112], Solamargine [113]	TUG1	miR-384 (nsopharynx ca) [114]	↑migration	PDCD6IP
	Curcumin [115]	LINC-ROR	miR-145 (endometrial ca) [116]	↓differentiation	X
	Curcumin [117], Sulforaphane [118], Bharangin [105], Ginsenosides [105], Triptolide [119]	H19	miR-141-3p (gastric ca) [120]/miR-22-3p (gastric ca) [121], miR-152 (glioma) [122]	↑proliferation	X
	Sulforaphane [105]	LINC01116	miR-592 (leukemia) [123]	↑proliferation	X
	Solamargine [113]	HOTTIP	miR-216a-5p (prostate ca) [124]	↑proliferation	X
	Formononetin [57]	AFAP1-AS1	miR-195-5p (breast ca) [57]	↑proliferation	MYO5B, VPS4A
	Resveratrol [105]	MIR4435-2HG	miR-802 (melanoma) [125]	↑proliferation	RAB7A, SDC4
	Luteolin [105]	BANCR	miR-195-5p (pancreatic ca) [126]	↑proliferation	MYO5B, VPS4A
	Hyperoside, Baicalein [78,127]	CCAT1	miR-410-3p (glioma) [128]/miR-152-5p (lung ca) [129]	↑proliferation	RAB11A/SDC1, SMPD3
	Gemini Curcumin [130]	CCAT2	miR-424-5p (ovarian ca) [59]	↑proliferation	MYO5B, VPS4A
	Calycosin [105]	EWSAT1	miR-330-5p (cervical, nasopharynx ca) [131,132]	↑proliferation	CD34, PDCD6IP
	Berberis, Calycosin, Curcumin, 3,30-Diindolylmethane, Genistein, Silibinin [105]	HOTAIR	miR-217-5p (liver ca) [133]	↑proliferation	ATP9A, PDCD6IP, STEAP3
	Melatonin [134]	JPX	miR-362-3p (lung ca) [135]	↑proliferation	PDCD6IP
	Epigallocatechin-3-gallate [105]	LINC00511	miR-515-5p (gastric ca) [136]/miR-424-5p (liver ca) [137]	↑proliferation	RAB11A/MYO5B, VPS4A
	Betulinic acid [78], Quercetin [138], Resveratrol K [105]	MALAT1	miR-101-3p (colon ca) [139]/miR-129-5p (breast ca) [140]	↑proliferation	RAB27A/ATP9A, PDCD6IP, VPS4B
	Anisomycin [78,141,142]	MEG3	miR-21-5p (oral ca) [143]/miR-708-5p (colon ca) [144]	↑proliferation	MYO5B, RAB11A/SDC1
	Quercetin [138]	MIAT	miR-495-3P (leukemia) [145]/miR-330-5p (ovarian ca) [146]	↑proliferation	SDC4/CD34, PDCD6IP
	3,30-Diindolylmethane [105]	PCGEM1	miR-145-5p (prostate ca) [147]	↑proliferation	STAM
	Curcumin [78], Cardamonin [148]	PVT1	miR-30d-5p (gallbladder ca) [149]	↑proliferation	RAB11A
	Epigallocatechin-3-gallate [105]	SOX2-OT	miR-194-5p (gastric ca) [75]	↑proliferation	SDC4
	Gambogic acid [105]	SPRY4-IT1	miR-101-3p (colon ca) [150]	↑proliferation	RAB27A
	Huaier [78,151]	TP73-AS1	miR-329-3p (cervical ca) [152]	↑proliferation	PDCD6IP
	Curcumin [105], Usnic acid [153]	UCA1	miR-26a-5p (gastric ca) [154]/miR-144-3p (lung ca) [155]	↑proliferation	RAB11A/PDCD6IP, SMPD3, VPS4B
	Atractylenolide II [156], Platycodin D [157]	XIST	miR-200b-3p (liver ca) [158]	↑proliferation	PRKN, STAM
	Silibinin [105]	ZFAS1	miR-135a-5p (nasopharynx ca) [159]	↑proliferation	PDCD6IP, SDCBP
**Upregulation**	Curcumin [105]	TUSC7	miR-224-5p (esophagus ca) [160]/miR-10a-5p (glioblastoma) [161]	↓chemoresistance	ATP9A/SDC1, SMPD3
	Baicalein [162]	PAX8-AS1	miR-96-5p (thyroid ca) [163]	↓proliferation	MYO5B, RAB27A, RAB7A
	Bharangin, Curcumin, Gambogic acid [105], Corylin [164]	GAS5	miR-222-3p (leukemia, gastric ca) [165,166]/miR-196a-5p (breast ca) [167]	↓proliferation	X
	Ginsenosides [105]	STXBP5-AS1	miR-96-5p (cervical ca) [168]	↓proliferation	MYO5B, RAB27A, RAB7A
	Resveratrol [105]	PCAT29	miR-494 (lung ca) [169]	↓proliferation	X

↑, enhance; ↓, inhibit. The predicted sponging miRNAs of lncRNAs were retrieved from the lncTarD 2.0 database. Predicted exosomal processing gene targets of sponging miRNAs were retrieved from the miRDB database (retrieval date: 1 March 2023). X indicates that no potential target is available in the miRDB database.

**Table 4:** Exosome-processing-target-centric view of predicted sponging miRNAs and natural-product-modulated lncRNAs.

Exosome Processing	Sponging miRNAs	lncRNAs	Natural Products	Exosome Processing	Sponging miRNAs	lncRNAs	Natural Products
ATP9A	217-5p	HOTAIR	↓Berberis, Calycosin, Curcumin, 3,30-diindolylmethane, Genistein, Silibinin	PDCD6IP	330-5p	EWSAT1	↓Calycosin
	129-5p	MALAT1	↓Betulinic acid, Quercetin, Resveratrol		217-5p	HOTAIR	↓Berberis, Calycosin, Curcumin, 3,30-diindolylmethane, Genistein, Silibinin
	224-5p	TUSC7	↑Curcumin		362-3p	JPX	↓Melatonin
CD34	330-5p	EWSAT1	↓Calycosin		129-5p	MALAT1	↓Betulinic acid, Quercetin, Resveratrol
		MIAT	↓Quercetin		330-5p	MIAT	↓Quercetin
MYO5B	195-5p	AFAP1-AS1	↓Formononetin		329-3p	TP73-AS1	↓Huaier
		BANCR	↓Luteolin		384	TUG1	↓Polydatin, Solamargine
	424-5p	CCAT2	↓Gemini Curcumin		144-3p	UCA1	↓Curcumin, Usnic acid
		LINC00511	↓epigallocatechin-3-gallate		135a-5p	ZFAS1	↓Silibinin
	21-5p	MEG3	↓Anisomycin	SDC1	152-5p	CCAT1	↓Hyperoside, Baicalein
	96-5p	PAX8-AS1	↑Baicalein		708-5p	MEG3	↓Anisomycin
		STXBP5-AS1	↑Ginsenosides		10a-5p	TUSC7	↑Curcumin
PRKN	200b-3p	XIST	↓Atractylenolide II, Platycodin D	SDC4	802	AK001796	↓Resveratrol
RAB11A	410-3p	CCAT1	↓Hyperoside, Baicalein		495-3p	MIAT	↓Quercetin
	515-5p	LINC00511	↓epigallocatechin-3-gallate		194-5p	SOX2-OT	↓epigallocatechin-3-gallate
	21-5p	MEG3	↓Anisomycin	SDCBP	135a-5p	ZFAS1	↓Silibinin
		NBR2	↑Curcumin	SMPD3	152-5p	CCAT1	↓Hyperoside, Baicalein
	30d-5p	PVT1	↓Curcumin, Cardamonin		10a-5p	TUSC7	↑Curcumin
	26a-5p	UCA1	↓Curcumin, Usnic acid		144-3p	UCA1	↓Curcumin, Usnic acid
RAB27A	101-3p	MALAT1	↓Betulinic acid, Quercetin, Resveratrol	STAM	128-3p	HOTTIP	↓Solamargine
	96-5p	PAX8-AS1	↑Baicalein		145-5p	PCGEM1	↓3,30-diindolylmethane
	101-3p	SPRY4-IT1	↓Gambogic acid		200b-3p	XIST	↓Atractylenolide II, Platycodin D
	96-5p	STXBP5-AS1	↑Ginsenosides	STEAP3	217-5p	HOTAIR	↓Berberis, Calycosin, Curcumin, 3,30-diindolylmethane, Genistein, Silibinin
RAB7A	802	AK001796	↓Resveratrol	VPS4A	195-5p	AFAP1-AS1	↓Formononetin
	96-5p	PAX8-AS1	↑Baicalein			BANCR	↓Luteolin
		STXBP5-AS1	↑Ginsenosides		424-5p	CCAT2	↓Gemini curcumin
						LINC00511	↓epigallocatechin-3-gallate
				VPS4B	128-3p	HOTTIP	↓Solamargine
					129-5p	MALAT1	↓Betulinic acid, Quercetin, Resveratrol
					144-3p	UCA1	↓Curcumin, Usnic acid

↑ and ↓ indicate the enhancement and inhibition of the lncRNA expression by natural products, respectively. This table provides exosomal-processing-target-centric connections to sponging miRNAs and natural-product-modulated lncRNAs. Exosomal processing targets include exosomal assembly and secretion. All the information is derived from Table 3, but only the information showing exosomal process targets is plotted. Sponging miRNAs and exosomal processing targets were predicted using the lncTarD and miRDB.
